# Gender inequality and national gender gaps in overconfidence

**DOI:** 10.1371/journal.pone.0249459

**Published:** 2021-04-15

**Authors:** Hayk Amirkhanyan, Michał Wiktor Krawczyk, Maciej Wilamowski

**Affiliations:** Faculty of Economic Sciences, University of Warsaw, Warsaw, Poland; Heidelberg University, GERMANY

## Abstract

Using a large dataset of marathon runners, we estimate country- and gender-specific proxies for overconfidence. Subsequently, we correlate them with a number of indices, including various measures of gender equality. We find that in less gender-equal countries both males and females tend to be more self-confident than in more equal countries. While a substantial gender gap in overconfidence is observed, it only correlates with some sub-indices of gender equality. We conclude that there is likely a weak relationship between OC gender gap and gender inequality.

## Introduction

Studies in judgment and decision-making sometimes find systematic differences between genders. It is important to understand how these differences may be shaped by malleable, cultural factors (such as differentiated patterns of socialization of boys and girls) and how they may contribute to inequalities in wealth and power. One way to establish this would be to run large international studies to find out if size of gender gap differs between countries and, if so, what are its correlates. Unfortunately, most laboratory studies are conducted with small, homogeneous samples representing a handful of Western, educated, industrialized, rich and democratic (aka WEIRD, [1]) countries.

In this project we focus on the often-observed gender gap in overconfidence (OC), specifically the tendency to overestimate one’s abilities compared to an objective benchmark (*overestimation*, see [2]). Using the proxy for OC based on pacing in marathons proposed by [3], we are able to measure gender gap in OC in around 70 countries. We then correlate it with a battery of country-specific indicators.

## Related literature

This project is related to several strands of literature. First, there are studies that investigate gender gap in overestimation in various field contexts. For example, Bengtsson, Persson and Willenhag [4] study gender differences in OC in a university exam setting. The exam consists of four questions and an additional fifth one for those students, who think they have “very good” answers for the first four. The authors find that, among the students who answered all initial four questions correctly, males were slightly more likely to answer the additional question (87.1 vs. 83.8%). This effect is stronger for the low ability students’ sub-sample. Similar results are reported for exams with penalty points for incorrect answers, which make less confident students–predominantly females–skip too many questions [5, 6]. These findings are foreshadowed by Lundeberg, Fox and Punćochaŕ [7], who directly ask students about their confidence level regarding their answers to university exam questions. The results reveal that although both genders exhibit OC, (undergraduate) males particularly tend to be overly confident when giving an incorrect answer. Such pattern tends to emerge also in country/region-level comparisons. Particularly, while majority of the studied regions show OC, higher OC is primarily associated with the regions that have low scores on cognitive skills test [8].

While numerous studies suggest that both genders tend to be OC, Dahlbom et al. [9] find high-school girls, but not boys, to be under-confident about the upcoming math test score. One reason for this could be that math tasks are often labelled as masculine. Relatedly, Beyer and Bowden [10] report lower female self-assessment and confidence in masculine but no gender difference in neutral and feminine tasks.

Gender gap in OC has implications in investment and financial decisions. Using S&P 1500 companies’ acquisition bids data, Levi, Li and Zhang [11] argue that lower overestimation by female directors results in fewer bids and lower bid premiums paid, thus creating shareholder value. Moreover, Barber and Odean [12] analyze around 35,000 households’ common stocks trading data and report that, consistent with the theory, males trade 45 percent more than females, which reduces their yearly net returns by around 2.6 percent as compared to around 1.7 percent decrease for females.

Johnson et al. [13] study OC behavior in wargames, additionally collecting testosterone samples. Subjects of the experiment play the role of the leader of a state in a computerized strategy game and may employ various strategies (negotiating, starting a war, etc.). Apart from confirming that subjects tend to be OC about their success (males more so than females) the authors report that higher OC in general is associated with more attacks (males attack significantly more often than females) and that testosterone levels correlate significantly with OC.

As it comes to gender differences, Lenney [14] suggests that females’ lower self-confidence depends (among other attributes like feedback, social comparison or assessment) on the sex-type of the task (i.e. masculine/feminine) and whether the setting is competitive. Lirgg [15] runs a meta-analysis of papers, which use physical activity tasks, to confirm this argument–in masculine and neutral tasks females tend to show lower self-confidence. More recent studies also report that males generally tend to record higher overplacement [16–19]. Bordalo et al. [17], for instance, investigate the role of task difficulty and stereotypes (about own ability and the ability of the opposite sex) in misestimation of own and others’ ability and find generally lower self-confidence in females and higher self-confidence in males, but solely in masculine tasks. More specifically, they find the following: the difficulty of a task increases overestimation of own and others’ ability. Moreover, stereotypes lead females to be underconfident about own abilities in masculine tasks. Stereotypes about others lead both genders to overestimate the ability of females compared to males in feminine tasks and underestimate it in masculine tasks.

Obviously, the studies most directly related to our paper are those that investigate pacing in marathons, generally finding that females pace more evenly, whereas males appear to be overly confident by starting too fast [3, 20–26]. An exception is the study by Deaner, Addona and Hanley [27] who find no significant gender difference in marathon pacing. Smyth [25] focuses on pacing in the beginning and in the end of a marathon. He finds that starting or finishing too fast may lead to worse finish times and that females generally tend to pace more evenly than males. Hubble and Zhao [24] too, find that uneven marathon pacing leads to worse performance. They use a finish time predictions database to also show that males (due to OC, they propose) tend to overestimate their ability compared to females and that males tend to slow down more in the later parts of the run. Both Trubee et al. [22] and Santos-Lozano et al. [21] compare the pacing of professional (top finishers) and amateur runners and find that top runners tend to have more stable speed than amateurs. Also, while the latter paper reports higher speed variability among males relative to females both for top and amateur runners, the former paper finds such a gender difference in pacing (which is larger in the case of the race in a hot weather) only among amateur runners.

Our paper is also closely related to studies investigating OC (but not specifically OC gender gaps) across countries. These have reported higher OC in China and India compared to the UK and the US [3, 28, 29]. Acker and Duck [28] conduct a stock market simulation involving 111 bachelor students mainly from UK and China. They observe Chinese students to be more OC than UK students (also, they find no correlation between different measures of OC, i.e. overestimation and overplacement). These authors also report that British males tend to be more OC than British females but find no gender difference among Chinese students. Our previous study using a much larger dataset of marathon runners also confirms that Asians tend to be relatively more OC than Westerners [3]. Moore, Dev and Goncharova [29] conduct two online studies involving participants from the US, the UK, Hong Kong and India to explore the cultural dimensions of the three types of OC: overestimation, overplacement and overprecision. They find higher overestimation among Indian participants, but no difference of overplacement and overprecision across all cultural groups. In addition, Giacomin, Janssen and Shinnar [30] examine entrepreneurial optimism and OC of university students and faculty in USA, Spain and India and report that US students tend to be more optimistic (but not OC) than Spanish and Indian students.

The crux of our approach is to measure gender gap at national level and link it to various country-specific variables. Several previous studies follow this path, but focusing on topics other than overconfidence. Nosek et al. [31], for instance, link gender-science stereotypes with sex differences in performance in science and math. They find that country-level stereotypes (relating math and science more with males than females) explain the gender difference in 8^th^ grade math and science performance. Another finding is that gender difference in math tends to disappear in more gender-equal countries [32]. Besides, female adolescents are more inspired by national gender-equal ideology and form higher educational expectations than males, who actually downgrade their expectations [33].

Some studies investigate the link between country-level gender equality and subjective health and well-being [34–37]. The results, however, seem to be mixed. While [36] and [34] find some evidence that country-level gender inequality tends to correlate with gender gap in subjective well-being and health, [35] and [37] do not find such a relation. Interestingly, the latter study reports higher female happiness and life satisfaction in countries with high/low proportions of Muslims/Catholics and no communism past. There is also limited evidence on the link between country-level gender empowerment and gender differences in emotions [38].

## The method

Our previous paper [3] proposed a proxy for OC in marathon runners: the Slowdown measure, defined as

Slowdown=timeatfinish–2*(21kmsplit),

whereby the 21km split is the time it took the runner to cover the first half of the distance. The Slowdown is thus zero if the runner keeps the constant pace throughout the race, which is, as physiology literature suggests (see e.g. [39]), approximately the optimal strategy (in case of a flat course and non-extreme weather conditions), regardless of the gender of the runner. As highlighted earlier in the literature review, starting or finishing the race too fast is associated with a worse performance (see e.g. [24, 25]). It would be reasonable to assume that most (if not all) marathon runners know this, given that almost all marathon forums and books urge not to start the race too fast. In practice, most runners start relatively fast and then their pace deteriorates, a positive Slowdown. Of course, this could result from minor injuries and other unpredictable events, but only in a small fraction of runners, certainly not majority of them. Physiological factors could also potentially explain some part of demographic differences observed (notably that males slow down more than females do, see [23] for a short discussion). However, rational decision makers, benefitting from extensive trials and feedback, should be able to correct for their knowable physical weaknesses, especially given that modern electronic devices, in addition to on-site pacesetters, make keeping optimal (constant) pace easier. Most tellingly, our results [3] showed that the tendency to slow down correlates highly (0.71) with forecast error: runners who make overly self-confident forecasts, tend to start too fast. Relatedly, both measures of OC–declared and revealed–tend to be similarly affected by demographic and psychological factors. This is to be expected: aiming at a constant pace, marathoners run the first kilometers–and as long as they can–at the pace they believe they could keep throughout the race. Usually though, they are proved wrong, their overconfidence showing not on the in the discrepancy between predicted and actual time but also between the pace in the last vs. the first half of the race. These observations boost our confidence that Slowdown belongs to the domain of judgment and decision making and, specifically, that it is a valid proxy for OC indeed.

### Data

We take the data from 40 marathons at six different locations, including Chicago, London and New York, see S1 Table for the complete list of events covered (dataset, which also includes short-distance race results, is freely available in “research data” section of [26]). In total, we have more than 1,145,000 results, 38% of which are for females. It may be worth mentioning that the differences in country-level shares of females in the data are not very large: 51 countries out of 70 have male share between 0.6 and 0.8, of which 33 countries are between 0.67 and 0.77; minimum share of males is 0.4 (Bahrain) and maximum is 0.87 (Morocco). Runners’ mean (median) age is 39.6 (39), they come from 69 countries with 56 countries being represented by at least 200 runners each. Nearly all of them are amateurs, though some spend many hours every week running and have participated in dozens of organized running events before. Obviously, our sample of marathon runners is not random. In particular, it is possible that more OC individuals are more likely to sign up. However, several arguments make us believe that the selection issue does not invalidate our findings.

First, highly overconfident runners who start fast and later slow down excessively, are more likely to experience injuries and to perform much below expectations, both of which would make them less likely to participate in future events. Hence, one could argue that extreme OC would not be a likely feature of marathon runners. Second, it is worth noting that in this study we focus on directional but not quantitative country-specific gender gaps. So, even if there is overall selection on OC, it is less likely to disprove the patterns we observe in our results, unless we strongly believe to encounter country/gender specific differences in selection. For example, it would have to be the case that the selection on overconfidence is much stronger among French than American women (but not men) or among Canadian than Italian men (but not women) etc. We do not see very compelling reasons to believe in such complex patterns, although it is difficult to rule them out.

It may be possible that the selection among females is stronger than among males. Indeed, a recent report [40] reveals that throughout the world the share of women among all runners for the past 15 years is close to 45% on average, thus somewhat higher than in our dataset or in most other marathons. If a stronger female selection existed, one would expect them to be more OC than males, which is clearly the opposite of what we see in our data. In any case, it would seem that our naturally occurring sample is more representative (i.e. more diverse in terms of education, employment, age) anyway than most laboratory studies on OC, in which the participants are volunteering students from selected research universities.

Individual pacing may depend on a number of features other than gender and nationality. To identify country-specific gender differences in our proxy for OC, we regress Slowdown on the male dummy and country dummies interacted with both gender dummies (taking the US, which is represented by the largest number of runners by far, as base category), additionally controlling for age, race-specific dummies and the 21-km split time (see S2 Table for the country estimates and S1 Fig for a map). The resulting estimate for the variable “male” can be understood as gender gap in OC in the US: American men on average slow down by 341 seconds more than American women do. The estimates of country-specific OC gender gap are easily calculated by adding the estimated coefficient for the relevant country*male dummy to that of the (US) male dummy and subtracting the estimated coefficient for the country*female dummy. For example, given the estimate for France*male (-101) and France*female (-117) we conclude that the French tend to be less OC than the Americans but the French OC gender gap is slightly larger than that in the US, equaling 341+(−101)−(−117) = 357 seconds.

We then correlate our country-specific estimates of OC gap with a number of national measures taken from earlier literature. We obtain data sets from a number of sources and group them in five categories: gender equality indices, entrepreneurship, culture, economic indicators and other (see S3 Table for all data sources, definitions and years).

First, we hypothesize that country-level gender gaps in OC could be linked to each country’s level of gender inequality. This is because greater gender equality in a country could boost females’ confidence relative to males’, which would in turn further strengthen equality. The other side of the coin is that gender difference in OC could play a role in perpetuating gender inequality. We thus add a number of measures of gender equality, including the Gender Equity Index (GEI, components: education, empowerment and economic participation) published by Social Watch, the Gender Inequality Index (GII, components: health, empowerment (secondary education and politics) and labor market participation) published by the United Nations Development Program and the Global Gender Gap Index (GGGI, sub-indices such as educational attainment, political empowerment, economic participation and opportunity are included in our analyses) published by World Economic Forum. We expect lower OC gender gaps in generally more gender-equal countries.

Second, following the literature that finds OC to be a determinant of starting a business, we include in our analysis, among others, country-level female/male ratio of the number of entrepreneurs and Total Entrepreneurial Activity (TEA), fraction of firms with female CEO and fraction of permanent full-time female workers. We expect to observe positive correlation between gender gaps in OC and gender gaps in entrepreneurship.

Third, we include several cultural variables that might be related to national gender differences in OC. One of such variables is the type of a language spoken in a country. Prewitt-Freilino, Caswell and Laakso [41] find higher gender inequality in countries where a *gendered* language (such as Russian, Spanish, Hindi, or German, for example) is spoken, which means that nouns are always assigned a feminine or masculine form. Likewise, higher gender inequality has been linked to greater religiosity [42]. Moreover, because there is evidence that overconfidence might lead to military conflicts (e.g. [13]), among cultural indicators we also include variables such as proportion of people in a country considering rule by military or by one strong leader as good for the country. Besides, we include the fraction of people agreeing that “a wife must always obey her husband” (regrettably, these are available only for a subset of our countries; source: PEW Forum on Religion and Public Life).

Fourth, economic indicators such as gross domestic product (GDP), GINI index, human development index (HDI) and unemployment rate are included in our analyses. We would expect positive correlation between economic inequality (i.e. GINI) and gender gaps in OC. As there is not much research (if any) on the relation between major macroeconomic indicators and country’s mean OC, we include those variables to check for any significant correlation.

Finally, we link gender gaps in OC with several other variables. Stoet and Geary [43] paradoxically find higher gender inequality among STEM (science, technology, engineering and math) graduates in countries with higher gender equality. Hence, in our analyses we include their measure of proportion of females among STEM graduates. We also check for a correlation between our measure of OC and the level of militarization in a given country. Namely, we use Global Militarization Index (GMI) and its sub-indices Military Expenditure Index Score and Military Personal Index introduced by Bonn International Center for Conversion, as wars have been linked to (male) overconfidence. In addition, we account for crime statistics, including in our analyses the rate of homicide, incarceration, serious assault and robbery. The logic for this inclusion is that at least some individuals committing serious crimes and ending up in jail (vast majority of whom happen to be male) may have overestimated their chances of getting away with it. We thus expect higher gender gap in OC in countries with higher crime rates.

## Results

We calculate (Spearman) correlations between our measure of OC and the indicators listed above. Table 1 presents the correlation coefficients, *p* values and *q* values (i.e. corrected–under each of our five categories separately–*p* values based on Holm’s method) for the OC gender gap, male and female OC (columns 1–3, 4–6 and 7–9 respectively). Although after Holm’s correction many coefficients become insignificant, we believe that reporting both *p* and *q* values and briefly discussing the results would be beneficial for the reader to get deeper insights from the analyses.

**Table 1:** Correlation coefficients between country-level indicators and gender gap in OC, male and female OC.

Nr	Variable	Male OC—Female OC	*p* value	*q* value	Male OC	*p* value	*q* value	Female OC	*p* value	*q* value
**Gender equality indices**		**1**	**2**	**3**	**4**	**5**	**6**	**7**	**8**	**9**
1	GGGI	-0.14	0.267	1	-0.31	**0.014**	0.109	-0.33	**0.008**	**0.060**
2	GGGI_Econ_opp	-0.27	**0.034**	0.306	-0.14	0.265	0.794	-0.04	0.765	1
3	GGGI_Educ	-0.07	0.609	1	-0.23	**0.067**	0.403	-0.21	0.101	0.404
4	GGGI_Politics	0.15	0.229	1	-0.26	**0.041**	0.290	-0.46	**0.000**	**0.001**
5	GII2010	0.26	**0.037**	0.306	0.47	**0.000**	**0.001**	0.3	**0.015**	**0.092**
6	BIGI	0.03	0.809	1	-0.06	0.626	1	-0.02	0.862	1
7	AADP	0.15	0.249	1	0.34	**0.006**	**0.050**	0.34	**0.006**	**0.058**
8	abs_Basic_Educ	0.33	**0.008**	**0.082**	0.21	**0.089**	0.444	0.01	0.940	1
9	abs_Healthy_Life	-0.36	**0.004**	**0.041**	0	0.987	1	0.32	**0.011**	**0.074**
10	abs_Life_Satis	-0.05	0.711	1	0.19	0.143	0.571	0.25	**0.048**	0.240
11	GEI	-0.26	**0.039**	0.306	-0.48	**0.000**	**0.001**	-0.37	**0.003**	**0.027**
**Entrepreneurship**										
12	Fear_of_failure	-0.05	0.754	1	0.05	0.773	1	0.15	0.384	1
13	Entrep_intentions	0.22	0.190	1	0.17	0.303	1	-0.05	0.758	1
14	Early_stage_TEA	0.1	0.570	1	-0.03	0.882	1	-0.22	0.192	1
15	F_M_TEA	0.05	0.776	1	0.11	0.510	1	-0.11	0.516	1
16	F_M_Opp_TEA	0.19	0.271	1	-0.02	0.928	1	-0.27	0.100	1
17	Entrep_Good_Career	-0.27	0.106	1	0.04	0.837	1	0.14	0.415	1
18	Employers_F_M	-0.19	0.250	1	-0.09	0.594	1	0.12	0.475	1
19	Own_F_M	0.28	**0.098**	1	-0.39	**0.018**	0.236	-0.62	**0.000**	**0.001**
20	Perc_has_F_owner	0.2	0.222	1	0.16	0.316	1	-0.05	0.774	1
21	Perc_F_owner	-0.04	0.847	1	0.2	0.293	1	0.17	0.379	1
22	Perc_F_manager	0.01	0.930	1	0.41	**0.010**	0.145	0.33	**0.042**	0.550
23	Perc_full_time_F	-0.14	0.401	1	0.18	0.257	1	0.31	**0.052**	0.630
24	Perc_full_time_F_prod	-0.09	0.591	1	0.2	0.233	1	0.24	0.146	1
25	Perc_full_time_F_non_prod	0.11	0.513	1	0.01	0.976	1	-0.1	0.541	1
**Culture**										
26	WifeObey_Agree	-0.7	**0.025**	0.100	0.16	0.651	0.651	0.92	**0.000**	**0.001**
27	Strong_leader_good	0.3	0.119	0.357	0.63	**0.000**	**0.001**	0.61	**0.000**	**0.001**
28	Military_good	0.47	**0.010**	**0.050**	0.44	**0.017**	**0.050**	0.26	0.180	0.360
29	Lang_type^a^	-0.1	0.673	0.673	0.13	0.130	0.259	0.02	0.419	0.419
30	Perc_NonRel	-0.12	0.328	0.657	-0.5	**0.000**	**0.000**	-0.44	**0.000**	**0.001**
**Economic indicators**										
31	GINI	0.28	**0.026**	0.103	0.28	**0.023**	**0.045**	0.03	0.823	0.823
32	GDP_percap_ppp	-0.27	**0.028**	0.103	-0.3	**0.014**	**0.043**	-0.19	0.117	0.350
33	GDP_percap_nominal	-0.22	**0.069**	0.137	-0.4	**0.001**	**0.004**	-0.32	**0.008**	**0.041**
34	Unemploy	0.05	0.723	0.723	-0.18	0.149	0.149	-0.14	0.258	0.516
35	HDI	-0.29	**0.020**	**0.098**	-0.41	**0.001**	**0.004**	-0.28	**0.023**	**0.090**
**Other**										
36	STEM	-0.14	0.394	0.787	0.26	0.113	0.793	0.37	**0.026**	0.140
37	GMI	-0.4	**0.001**	**0.008**	-0.08	0.549	1	0.14	0.264	0.792
38	Military_Expenditure	-0.23	**0.069**	0.347	0.16	0.197	0.793	0.29	**0.020**	0.140
39	Military_Personnel	-0.3	**0.017**	0.122	0.04	0.769	1	0.18	0.151	0.603
40	homicide	0.25	**0.049**	0.293	0.2	0.119	0.793	0.01	0.929	0.929
41	prison_rate	-0.01	0.932	0.932	0.2	**0.094**	0.754	0.13	0.304	0.792
42	serious_assault_rate	0.15	0.241	0.722	-0.06	0.621	1	-0.29	**0.020**	0.140
43	robbery_rate	0.22	**0.086**	0.347	-0.2	0.120	0.793	-0.51	**0.000**	**0.000**

Significance below 10% is highlighted in bold. *q* values represent corrected *p* values using Holm’s method (-qqvalue- package in stata). The correction is done for each category separately, i.e. gender equality indices, culture, etc.

^a^ Because language type is binary (we consider gendered and natural language types as defined in the original paper) we report point-biserial correlation.

### Gender gap in OC

We start with the discussion of the significant correlations with the gender gap (Table 1, column 1). Among gender equality indices we find correlation for the Gender Equity Index (GEI) and Gender Inequality Index (GII). The negative correlation coefficient for GEI (higher value = higher equality) means that higher gender equality is associated with lower gender gap in OC. Likewise, the positive coefficient of GII (higher value = higher inequality) means an association between higher gender inequality and higher gender gap in OC. This is in line with our initial hypothesis that gender gap in OC might perpetuate gender inequality. However, these effects are not significant under our (conservative) correction for multiple comparisons. Global Gender Gap Index (GGGI) and Basic Index of Gender Inequality (BIGI, [44]), which is a simpler version of the former, on the other hand, seem to be unrelated to our OC measure.

However, a few sub-indices of BIGI and GGGI seem to be correlated with OC gap. Particularly, the absolute value (own calculations, higher value = higher inequality) of Basic Education component of BIGI (row 8) has a positive correlation coefficient, meaning that higher gender gap in OC is associated with higher gender inequalities in basic education. It would be difficult to provide a comprehensive explanation for this result. First, the direction of causality is uncertain. In other words, it is unclear whether OC gender gap leads to gender gap in basic education or vice versa. Besides, the link between OC gender gap and basic education gender gap might be indirect. Perhaps there are several other factors, including overall gender inequalities, that affect one or the other. By contrast, we find no significant correlation between STEM (see the “other” category) and our OC gap measure. Surprisingly, a significant negative coefficient is observed for absolute value (own calculation, higher value = higher inequality) of Healthy Life Expectation component of BIGI index. In other words, higher gender difference in healthy life expectation seems to be related to lower gender gap in OC. This seems to be driven by female OC. In other words, females seem to “catch up” with male OC in countries with higher gaps in healthy life expectancy (in our results healthy life expectancy gap has zero correlation with male OC and a significant positive correlation with female OC; see under columns 4 and 7). Again, it is unknown whether (if at all) OC gender gap causes gender gaps in healthy life expectancy or vice versa. Also, likely there are several factors in play here. For instance, gender gap in healthy life expectation has been linked to higher alcohol consumption [44], which itself is linked to risky behaviors and OC [45].

Among economic indicators we see that economic inequality (GINI: higher value = higher inequality), GDP per capita and HDI (higher value = more developed) are correlated with our measure of OC gender gap, although not significantly so when Holm’s correction is applied. A positive coefficient of GINI indicates a higher gender gap in OC in more unequal countries. This result, again, may be due to significantly higher male OC (and nearly zero correlation with female OC, see columns 4 and 7). Further, higher HDI and GDP per capita seem to be associated with lower gender gap in OC. These results are not surprising given that GINI is negatively related with both HDI and GDP (in our dataset all pairwise correlation p values are lower than 0.007).

Among “other” variables we observe significant coefficients for militarization and homicide/robbery rates. Although, again, after Holm’s correction only Global Militarization Index (GMI) remains significant. To our surprise, we observe a negative coefficient for GMI. Higher values (i.e. higher militarization) of this variable correspond to lower gender difference in OC, yet we observe no significant correlation for male and female OC. By contrast, among cultural variables we see that gender gap in OC is positively related to fraction of people who think that rule by military would be good for their country (data available for 29 countries). This result seems to be driven primarily by male OC (see under columns 4 and 7). One explanation to this contrasting results could be that GMI is measured based on national statistics (i.e. sub-indices: military expenditures as percentage of GDP, etc.), which represent societal preferences *indirectly*, whereas Military_good is (like OC) measured at an individual level. Perhaps this is why the estimates for Military_good more closely resembles the previous findings that higher (male) OC is related to wars [13].

We find no significant correlation between gender gap in OC and of the remaining variables included in our analyses. Notably, we see no significant correlation between gender gap in OC and the variables in “entrepreneurship” category. Besides, language type, the fraction of non-religious people in a country and unemployment rate seem to be unrelated to the OC gap.

### Male and female OC

An alternative look at the data is to correlate country-level male and, separately, female OC with the country characteristics listed before (Table 1, columns 4 and 7). Below is a brief discussion of the correlation coefficients significant at 10% level (after correction for multiple tests). We find consistent correlation among all the gender equality indices. GEI is negatively correlated with the OC of both genders, meaning that in more equal countries both males and females tend to be less OC. Similarly, GII has a positive coefficient for both genders, meaning higher male and female OC in more unequal societies. BIGI index too, indicates higher male and female OC in countries with higher inequality. Namely, Average Absolute Deviation from Parity score (AADP), which takes a higher value for countries that deviate more from gender equality, has a positive correlation with male and female OC measures. Moreover, negative coefficients of GGGI and its sub-index Political Empowerment (female/male ratio of number of positions at parliament and ministerial level and number of years with a female head of state) show that higher equality is associated with lower OC for both sexes (though, the coefficients for males lose significance after Holm’s correction).

Similarly, turning now to “entrepreneurship” variables, a higher female/male ratio of the number of own-account workers (row 19; self-employed, who have not hired employees to work for them on a regular basis) seems to be associated with lower male and (especially) female OC. Assuming that hiring employees might be associated with higher confidence and risk-taking, this result might not be surprising: one possibility could be that lower (female) OC leads more females to be/stay own-account workers (and not to hire employees). Then again, this is just one possibility given that the direction of causality is unknown. By contrast, both males and females seem to be more OC in countries with higher shares of firms that have a female CEO (row 22), although, again, this result turns out not to be robust to Holm’s correction for multiple comparisons.

Interestingly, we find several significant correlates among cultural variables and economic indicators. For instance, we observe that the percent of non-religious people in a country is associated with lower male and female OC. It seems that the relation between religiosity and OC has received little (if any) attention among researchers. However, this finding seems to contradict (given the link between OC and risk-taking) earlier research reporting negative relation between religiosity and risk-taking (see e.g. [46, 47]). In addition, it seems that both sexes are more OC in countries (29 of them in our dataset) where more respondents state that rule by one strong leader would be good for their country. Besides, higher (nominal) GDP per capita and HDI both seem to be associated with lower male and female OC. A significant positive coefficient of GINI for (only) males indicates higher male OC in countries with higher economic inequalities. All in all, indicators suggest that economic development is associated with lower OC. Yet again, it is not clear which one of them (if any) causing the other. It is likely that they both reinforce each other: lower OC might lead to more efficient economic outcomes, which in turn might affect psychological traits, including OC levels. Nevertheless, language type and unemployment rates seem to have no correlation with male or female OC.

Finally, among “other” variables we observe higher female OC in countries with higher share of females among STEM graduates in tertiary education. Besides, higher female OC seems to be related to country’s military expenditure. While it seems reasonable that female OC and female share among STEM graduates are correlated, the relation between military expenditure and female OC appears to be less obvious. One possibility is that females in states with higher military expenditure feel more confident. On the other hand, serious assault and robbery rates are negatively correlated with female OC, while incarceration rate has a (marginally) positive correlation with male OC. Only the robbery rate remains significant after correcting for multiple comparisons using Holm’s method, with females being significantly less OC in states with higher robbery rate. One explanation could be that (male) crime diminishes female confidence.

## Conclusion

In this study we use a unique data set of around 1,145,000 results of marathon runners to measure gender difference in OC in around 70 countries. We correlate these country-level indicators with a number of variables measuring gender relations in several socio-economic dimensions, notably gender equality indices and measures of gap in the entrepreneurship, as well as cultural and economic indicators. Although gender gap in OC correlates consistently with a number of measures of gender inequality (more equal countries typically show more similar OC levels of males and females), these effects are not significant when corrected for multiple comparisons.

This does not mean that gender inequality is not related to gender-specific OC at all. Historically, female emancipation meant, among many other things, that women picked up some of the men’s bad habits. Smoking represents a case in point here. In the US (and many other countries) smoking was very common among men and very rare among women around 1920 or so, but the gap essentially disappeared by 1980 [48]. This tectonic shift had much to do with the feminist movement [49, 50].

It would be troubling if we observed that in more gender-equal countries females tended to emulate males’ irrational OC (for example, this would suggest that problem gambling among females–typically being much lower than among males–could soon go up). Fortunately, we find the opposite to be true. In more gender-equal countries both genders tend to become somewhat less overconfident.

Again, our results are merely correlations. We do not have panel data to identify timing of the changes nor any sources of exogenous variation in any of our variables. Clearly, both of them would be highly desirable in future studies. Another interesting possibility for further research would be to investigate other dimensions of social inequity. For example, our previous research [3] found sizeable, nonmonotonic age effects, with the youngest and the oldest displaying highest OC. The question arises if country-specific age effects could be linked with measures of ageism, as well as society’s age structure.

## Supporting information

S1 Dataset (XLSX)

S1 Fig Gender gaps in OC in Europe. Made with Natural Earth. Free vector and raster map data @ naturalearthdata.com. Countries with no data are left white with no name tag. Europe is the only region with a large concentration of countries, for which we do have data.
(TIF)

S1 Table Summary statistics for the marathon data by location. (DOCX)

S2 Table Country male/female OC estimates. Estimates resulting from an OLS regression of Slowdown on the male dummy and country dummies interacted with both gender dummies (taking the US as base category), additionally controlling for age, race-specific dummies and the 21-km split time. A positive male OC could be understood as higher slowdown (OC) in seconds compared to males from the US; likewise, a positive female OC means higher slowdown compared to US females. Country-level gender difference in OC is calculated with the following formula: (US male OC + country male OC)—country female OC. Of course, including the US male OC and thus US gap (341) in all the national gaps makes no difference for the correlations, as it is a constant.
(DOCX)

S3 Table Data sources. ^a^ Health and Survival sub-index is not included in our analyses. ^b^ for the full list of BIGI variables see [44]. ^c^ we use two GDP per capita measures: 1. GDP_percap_ppp: GDP per capita, constant prices (purchasing power parity; 2011 international dollar), 2. GDP_percap_nominal: GDP per capita, current prices (US dollars). ^d^ Heavy Weapons Index score is not included in our analyses.
(DOCX)
