# Supplementary figures and images for: Gender inequality and national gender gaps in overconfidence
Source: PLoS One. 2021 Apr 15;16(4):e0249459. doi: 10.1371/journal.pone.0249459 (PMC8049476; doi:10.1371/journal.pone.0249459)

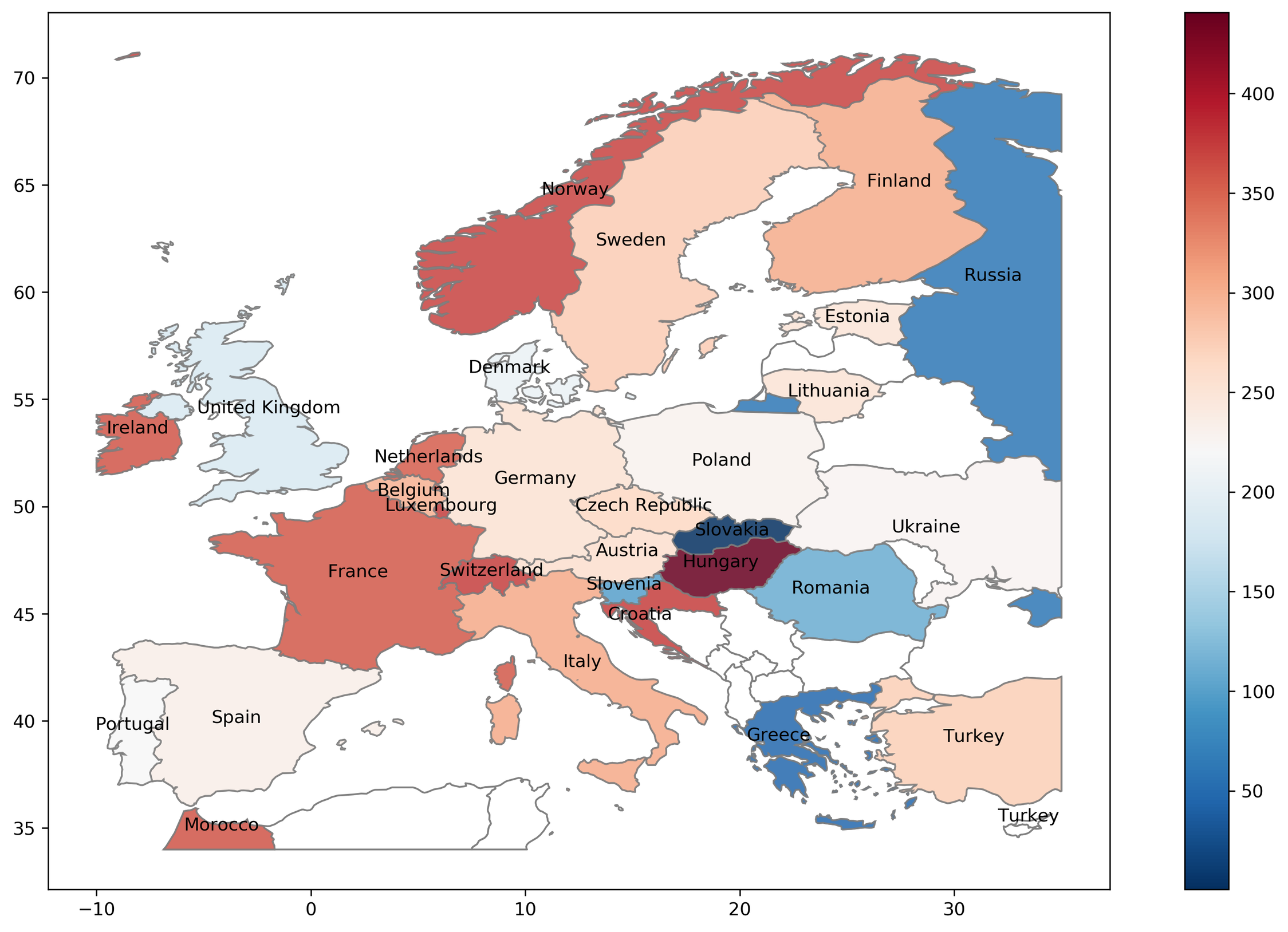

Supplement: S1 Fig — Made with Natural Earth. Free vector and raster map data @ naturalearthdata.com. Countries with no data are left white with no name tag. Europe is the only region with a large concentration of countries, for which we do have data. (TIF) [file pone.0249459.s002.tif]
