# Supplementary material for: Gender inequality and national gender gaps in overconfidence
Source: PLoS One. 2021 Apr 15;16(4):e0249459. doi: 10.1371/journal.pone.0249459 (PMC8049476; doi:10.1371/journal.pone.0249459)
Supplement: S1 Table — (DOCX) [file pone.0249459.s003.docx]

**S1 Table. Summary statistics for the marathon data by location.**

| **Location** | **# runners** | **# races** | **year** | |
| --- | --- | --- | --- | --- |
|  |  |  | **min** | **max** |
| London | 139,583 | 4 | 2010 | 2013 |
| Chicago | 490,995 | 16 | 1998 | 2013 |
| New York | 429,515 | 12 | 1999 | 2011 |
| Houston | 20,538 | 3 | 2012 | 2014 |
| Orlando | 38,394 | 2 | 2013 | 2014 |
| Hamburg | 26,168 | 3 | 2012 | 2014 |
