# Supplementary material for: Gender inequality and national gender gaps in overconfidence
Source: PLoS One. 2021 Apr 15;16(4):e0249459. doi: 10.1371/journal.pone.0249459 (PMC8049476; doi:10.1371/journal.pone.0249459)
Supplement: S2 Table — Estimates resulting from an OLS regression of Slowdown on the male dummy and country dummies interacted with both gender dummies (taking the US as base category), additionally controlling for age, race-specific dummies and the 21-km split time. A positive male OC could be understood as higher slowdown (OC) in seconds compared to males from the US; likewise, a positive female OC means higher slowdown compared to US females. Country-level gender difference in OC is calculated with the following formula: (US male OC + country male OC)—country female OC. Of course, including the US male OC and thus US gap (341) in all the national gaps makes no difference for the correlations, as it is a constant. (DOCX) [file pone.0249459.s004.docx]

**S2 Table. Country male/female OC estimates.**

| **Country** | **Continent** | **Male OC (comp. to US)** | **Female OC**  **(comp. to US)** | **Male OC - Female OC** |
| --- | --- | --- | --- | --- |
| Argentina | South America | -78.9 | -83.8 | 345.8 |
| Australia | Australia | -48.9 | 15.8 | 276.3 |
| Austria | Europe | -152.3 | -69.1 | 257.7 |
| Bahrain | Asia | -2.1 | 161.6 | 177.2 |
| Belgium | Europe | -226.3 | -183.8 | 298.5 |
| Bermuda | North America | -100.9 | 151.8 | 88.3 |
| Brazil | South America | -216.6 | -190.1 | 314.4 |
| Canada | North America | -191.1 | -178.4 | 328.3 |
| Chile | South America | -211.9 | -120.7 | 249.7 |
| China | Asia | -15.1 | -157.0 | 482.8 |
| Colombia | South America | -24.4 | -78.6 | 395.2 |
| Costa Rica | North America | -169.9 | -251.4 | 422.5 |
| Croatia | Europe | 32.2 | -0.7 | 373.9 |
| Czech Republic | Europe | -115.6 | -40.9 | 266.3 |
| Denmark | Europe | -166.7 | -31.4 | 205.8 |
| Dominican Republic | North America | 102.0 | -168.6 | 611.5 |
| Ecuador | South America | 147.3 | -58.0 | 546.2 |
| El Salvador | North America | 86.0 | 62.5 | 364.5 |
| Spain | Europe | -134.4 | -28.1 | 234.7 |
| Estonia | Europe | -98.2 | -6.8 | 249.6 |
| Ethiopia | Africa | 146.4 | 65.5 | 422.0 |
| Finland | Europe | -408.8 | -370.9 | 303.1 |
| France | Europe | -100.9 | -117.1 | 357.2 |
| United Kingdom | Europe | -1.8 | 153.4 | 185.7 |
| Germany | Europe | -286.3 | -198.5 | 253.2 |
| Greece | Europe | -195.1 | 99.2 | 46.7 |
| Guatemala | North America | -169.3 | 4.6 | 167.1 |
| Hong Kong | Asia | 61.5 | 129.8 | 272.7 |
| Hungary | Europe | 132.8 | 33.2 | 440.6 |
| India | Asia | 380.0 | 255.4 | 465.6 |
| Ireland | Europe | -6.5 | -24.9 | 359.4 |
| Iceland | Europe | -111.4 | -120.9 | 350.5 |
| Israel | Asia | -191.7 | -196.9 | 346.1 |
| Italy | Europe | 72.4 | 109.1 | 304.4 |
| British Virgin Islands | North America | 248.0 | 34.8 | 554.2 |
| Jamaica | North America | 335.1 | 43.5 | 632.6 |
| Japan | Asia | 198.8 | 119.8 | 420.1 |
| Kenya | Africa | 223.3 | 183.5 | 380.7 |
| Korea South | Asia | -6.1 | 29.2 | 305.7 |
| Lithuania | Europe | -0.6 | 89.0 | 251.4 |
| Luxembourg | Europe | -276.3 | -309.3 | 374.0 |
| Morocco | Africa | -44.1 | -63.5 | 360.5 |
| Malaysia | Asia | 159.6 | 163.7 | 336.9 |
| Mexico | North America | -51.2 | -77.3 | 367.1 |
| Netherlands | Europe | -235.3 | -248.6 | 354.2 |
| Norway | Europe | -61.9 | -92.0 | 371.1 |
| New Zealand | Australia | -126.9 | -149.5 | 363.6 |
| Panama | North America | -12.5 | 21.3 | 307.2 |
| Peru | South America | 100.3 | -31.9 | 473.2 |
| Philippines | Asia | 185.1 | 217.2 | 308.8 |
| Poland | Europe | -58.9 | 53.8 | 228.4 |
| Portugal | Europe | -126.5 | -6.2 | 220.7 |
| Puerto Rico | North America | 121.0 | 41.7 | 420.3 |
| Romania | Europe | 67.0 | 297.5 | 110.4 |
| South Africa | Africa | -27.0 | -43.5 | 357.5 |
| Russia | Europe | -185.0 | 94.8 | 61.2 |
| Singapore | Asia | 175.9 | 144.2 | 372.7 |
| Slovenia | Europe | -95.0 | 146.7 | 99.3 |
| Switzerland | Europe | -116.7 | -149.1 | 373.4 |
| Slovakia | Europe | -249.5 | 90.9 | 0.6 |
| Sweden | Europe | -152.9 | -88.4 | 276.5 |
| Thailand | Asia | 15.1 | 404.3 | -48.2 |
| Taiwan | Asia | 154.8 | 275.5 | 220.3 |
| Trinidad and Tobago | South America | 409.1 | 124.6 | 625.6 |
| Turkey | Asia | 19.5 | 85.9 | 274.6 |
| Ukraine | Europe | 47.3 | 162.0 | 226.4 |
| Uruguay | South America | -219.3 | -287.1 | 408.9 |
| Venezuela | South America | -2.0 | -32.5 | 371.6 |
| *United States (base)* | *North America* | *0* | *0* | *341* |

Estimates resulting from an OLS regression of Slowdown on the male dummy and country dummies interacted with both gender dummies (taking the US as base category), additionally controlling for age, race-specific dummies and the 21-km split time. A positive male OC could be understood as higher slowdown (OC) in seconds compared to males from the US; likewise, a positive female OC means higher slowdown compared to US females. Country-level gender difference in OC is calculated with the following formula: (US male OC + country male OC) ­­­- country female OC. Of course, including the US male OC and thus US gap (341) in all the national gaps makes no difference for the correlations, as it is a constant.
