# Supplementary material for: Gender inequality and national gender gaps in overconfidence
Source: PLoS One. 2021 Apr 15;16(4):e0249459. doi: 10.1371/journal.pone.0249459 (PMC8049476; doi:10.1371/journal.pone.0249459)
Supplement: S3 Table — a Health and Survival sub-index is not included in our analyses. b for the full list of BIGI variables see [44]. c we use two GDP per capita measures: 1. GDP_percap_ppp: GDP per capita, constant prices (purchasing power parity; 2011 international dollar), 2. GDP_percap_nominal: GDP per capita, current prices (US dollars). d Heavy Weapons Index score is not included in our analyses. (DOCX) [file pone.0249459.s005.docx]

**S3 Table.** **Data sources.**

| **Abbreviations** | **Name** | **Source** | **Years/countries covered** |
| --- | --- | --- | --- |
|  | **Gender equality indices** |  |  |
| GGGI | Global Gender Gap Index^a^ | World Economic Forum | 2007 |
| GGGI_Econ_opp | Economic Participation and Opportunity sub-index |  |  |
| GGGI_Educ | Educational Attainment sub-index |  |  |
| GGGI_Politics | Political Empowerment sub-index |  |  |
| GII | Gender Inequality Index | UNDP | 2010 |
| BIGI | Basic Index of Gender Inequality^b^ | [44] | average of 2012–2016 |
| GEI | Gender Equity Index | Social Watch  http://www.socialwatch.org/ | 2012 |
|  | **Entrepreneurship** |  |  |
|  | Global Entrepreneurship Monitor | Global Entrepreneurship Monitor https://www.gemconsortium.org/data/key-aps | 2018 |
| Fear_of_failure | Fear of failure rate (% 18-64 population perceiving good opportunities to start a business who indicate that fear of failure would prevent them from setting up a business) |  |  |
| Entrep_intentions | Entrepreneurial intentions (% 18-64 population (individuals involved in any stage of entrepreneurial activity excluded) who are latent entrepreneurs and who intend to start a business within three years) |  |  |
| Early_stage_TEA | Total early-stage Entrepreneurial Activity (TEA) (% 18-64 population who are either a nascent entrepreneur or owner-manager of a new business) |  |  |
| F_M_TEA | Female/Male TEA (% female 18-64 population who are either a nascent entrepreneur or owner-manager of a new business, divided by the equivalent percentage for their male counterparts) |  |  |
| F_M_Opp_TEA | Female/Male Opportunity-Driven TEA (% of those females involved in TEA who (i) claim to be driven by opportunity as opposed to finding no other option for work; and (ii) who indicate the main driver for being involved in this opportunity is being independent or increasing their income, rather than just maintaining their income, divided by the equivalent percentage for their male counterparts) |  |  |
| Entrep_Good_Career | Entrepreneurship as a Good Career Choice (% 18-64 population who agree with the statement that in their country, most people consider starting a business as a desirable career choice) |  |  |
|  | Entrepreneurship at a Glance 2017 | OECD  https://www.oecd-ilibrary.org/employment/entrepreneurship-at-a-glance-2017_entrepreneur_aag-2017-en | 2016 or latest available |
|  | # male and female employers |  |  |
|  | # male and female own account workers |  |  |
| Employers_F_M | ratio F/M employers | own calculations |  |
| Own_F_M | ratio F/M own account workers |  |  |
|  | World Bank enterprise surveys | World Bank https://www.enterprisesurveys.org/en/data/exploretopics/gender | latest available |
| Perc_has_F_owner | % firms with female participation in ownership |  |  |
| Perc_F_owner | % firms with majority female ownership |  |  |
| Perc_F_manager | % firms with female top manager |  |  |
| Perc_full_time_F | Fraction of permanent full-time workers who are female (%) |  |  |
| Perc_full_time_F_prod | Fraction of permanent full-time production workers who are female (%) |  |  |
| Perc_full_time_F_non_prod | Fraction of permanent full-time non-production workers who are female (%) |  |  |
|  | **Culture** |  |  |
| WifeObey_Agree | Wife Obey (% who agree with the statement, "a wife must always obey her husband") | PEW Forum on Religion and Public Life  https://www.pewforum.org/ | 2015-2016, 10 countries |
| Strong_leader_good | Strong Leader (% saying "a system in which a strong leader can make decisions without interference from parliament or the courts" would be a good way to govern the country) |  | 2017, 29 countries |
| Military_good | Military rule (% saying "the military rules the country" would be a good way to govern the country) |  |  |
| Lang_type | Language type (1 - Gendered, 2 - Natural) | [41] | 56 countries |
| Perc_NonRel | % non-religious (incl. atheists) people | Association of Religion Data Archives  http://www.thearda.com/internationalData/ | latest available, 69 countries |
|  | **Economic indicators** |  |  |
| GINI | GINI | World Bank and CIA.gov  https://data.worldbank.org/indicator/SI.POV.GINI https://www.cia.gov/library/publications/resources/the-world-factbook/fields/rawdata_223.txt | latest available |
| GDP | GDP^c^ | IMF https://www.imf.org/external/pubs/ft/weo/2019/02/weodata/weoselgr.aspx | 2017 |
| Unemploy | Unemployment rate |  |  |
| HDI | HDI | [44] (from BIGI database) | average of 2012–2015 |
|  | **Others** |  |  |
| STEM | STEM (the percentage of women among STEM graduates in tertiary education) | [43] | 2012-2015 |
| GMI | Global Militarization Index^d^ | Bonn International Center for Conversion (BICC)  https://gmi.bicc.de/ | 2018 |
| Military_Expenditure | Military Expenditure Index Score |  |  |
| Military_Personnel | Military Personal Index Score |  |  |
|  | Crime | UNODC |  |
| homicide | Homicide rates | https://dataunodc.un.org/crime/intentional-homicide-victims | 2010 |
| prison_rate | Prison population | https://dataunodc.un.org/crime | 2017 or latest available |
| serious_assault_rate | Serious Assault |  |  |
| robbery_rate | Robbery | https://dataunodc.un.org/data/crime/Robbery |  |

^a^ Health and Survival sub-index is not included in our analyses.

^b^ for the full list of BIGI variables see [44].

^c^ we use two GDP per capita measures: 1. GDP_percap_ppp: GDP per capita, constant prices (purchasing power parity; 2011 international dollar), 2. GDP_percap_nominal: GDP per capita, current prices (US dollars).

^d^ Heavy Weapons Index score is not included in our analyses.
